# Spatially modulated thermal light in atomic medium for enhanced ghost imaging

**DOI:** 10.1038/s41598-017-08374-3

**Published:** 2017-08-14

**Authors:** Mingtao Cao, Jinwen Wang, Xin Yang, Shuwei Qiu, Hong Gao, Fuli Li

**Affiliations:** Key Laboratory of Quantum Information and Quantum Optoelectronic Devices, Shaanxi Province, Xi’an Jiaotong University, Xi’an, 710049 China

## Abstract

Recent years have seen vast progress in image modulation based on atomic media, with potential applications in both classical optical imaging and quantum imaging regions. However, there have been few investigations of how thermal light images interact with an electromagnetically induced transparent medium. In this letter, we experimentally demonstrate pseudo-thermal light modulation on coherent population trapping conditions in ^87^ *Rb* vapor. By introducing the Laguerre-Gaussian beam as the control beam and the encoded speckle as the probe beam, we obtained sharper speckle patterns after the atom cell compared with that in free space. The spatially modulated thermal light was then used to enhance the image resolution in ghost imaging of which the resolution was enhanced by factor 3, since the ghost image resolution is heavily reliant on the speckle’s transverse coherent length. Our results are promising for potential applications in high resolution ghost imaging and image metrology, image processing and biomedical imaging.

## Introduction

Optical diffraction sets a fundamental limitation of the resolution in image formation for conventional optical devices and microscopy. Light carries information while images only form at certain locations, because each component acquires a different phase shift during propagation either in free space or in a medium, such that the image will be distorted even after propagating by only a few Rayleigh lengths owing to the superposition of all wave components^1^. Recently, image modulation in atomic media has attracted intense attention both in classical optical imaging and quantum imaging fields, because it can be used to significantly reduce the image diffraction and improve the image resolution for biomedical and other scientific applications^2, 3^. To obtain better image resolution, several approaches^4–6^ in which optical diffraction can be greatly suppressed or even eliminated by using atomic coherence effects have been suggested. The most promising approach to achieve better imaging modulation is based on coherent population trapping (CPT), in which the spatial structure of the images can be modulated more effectively by the susceptibility of the medium in such schemes^7–10^.

Actually, for coherent light image modulation in atomic media, great progress has recently been made by manipulating the atom susceptibility with the transverse distributed control beam^4, 5, 11^. There is experimental evidence that a better profile of the modulated image can be obtained due to the atomic modulation effect^12, 13^ and the image propagated without diffraction over a long distance. However, there have been few studies showing how the thermal light image interacts with electromagnetically induced transparent (EIT) medium, and it is of importance and interest to consider how thermal light interacts with an EIT medium and whether the intensity correlation and coherent area of the speckles can be improved during the CPT process. Therefore, it would be necessary to expand the capacity of modulating the coherent image to a thermal light image, such as a pseudo-thermal light speckle, which can be obtained by using the rotating ground glass. As is well-known, the speckle pattern in thermal light can be viewed as the multi-mode image, and it has been shown that the nature of the light can be well maintained in a four-wave mixing process^14^. Furthermore, it has also been proven that the image profile of a speckle pattern can be compressed in the CPT process^15^, which leads to much better resolution in ghost imaging (GI) experiments.

Ghost imaging is an imaging technique that has significant applications in optics imaging and quantum communication^16–18^. During the past decades, several techniques based on classical or quantum principles have been introduced to improve the resolution^19–22^; an alternative approach to these techniques is to change the speckle’s transverse coherent length by compressing and shaping the speckle profile. A smaller transverse coherence length means better GI resolution, and the compressed speckle pattern in an atomic medium would lead to new applications of GI.

In our previous work, we found that the nature of the thermal light was well maintained, and the compressed speckles from the atom cell improved ghost imaging resolution 1.4 times better than that in free space, and the image contrast was about 0.65. In this paper, we demonstrate that the ghost image resolution can be enhanced by using different spatial control beam in the EIT medium. In the experiment, we first generate the pseudo-thermal light speckle by sending a coherent light beam to the rotating ground glass, which was driven by a motor. Then the speckles were sending through the atom vapor on CPT condition. By coding different Laguerre-Gaussian (LG) charges onto the control beam, then applying them to the atom vapor, we found that the speckles profiles became 1.2 times smaller comparing with that of in free space. The compressed speckle made ghost imaging resolution 3 times better.

## Methods

**Figure 1:**
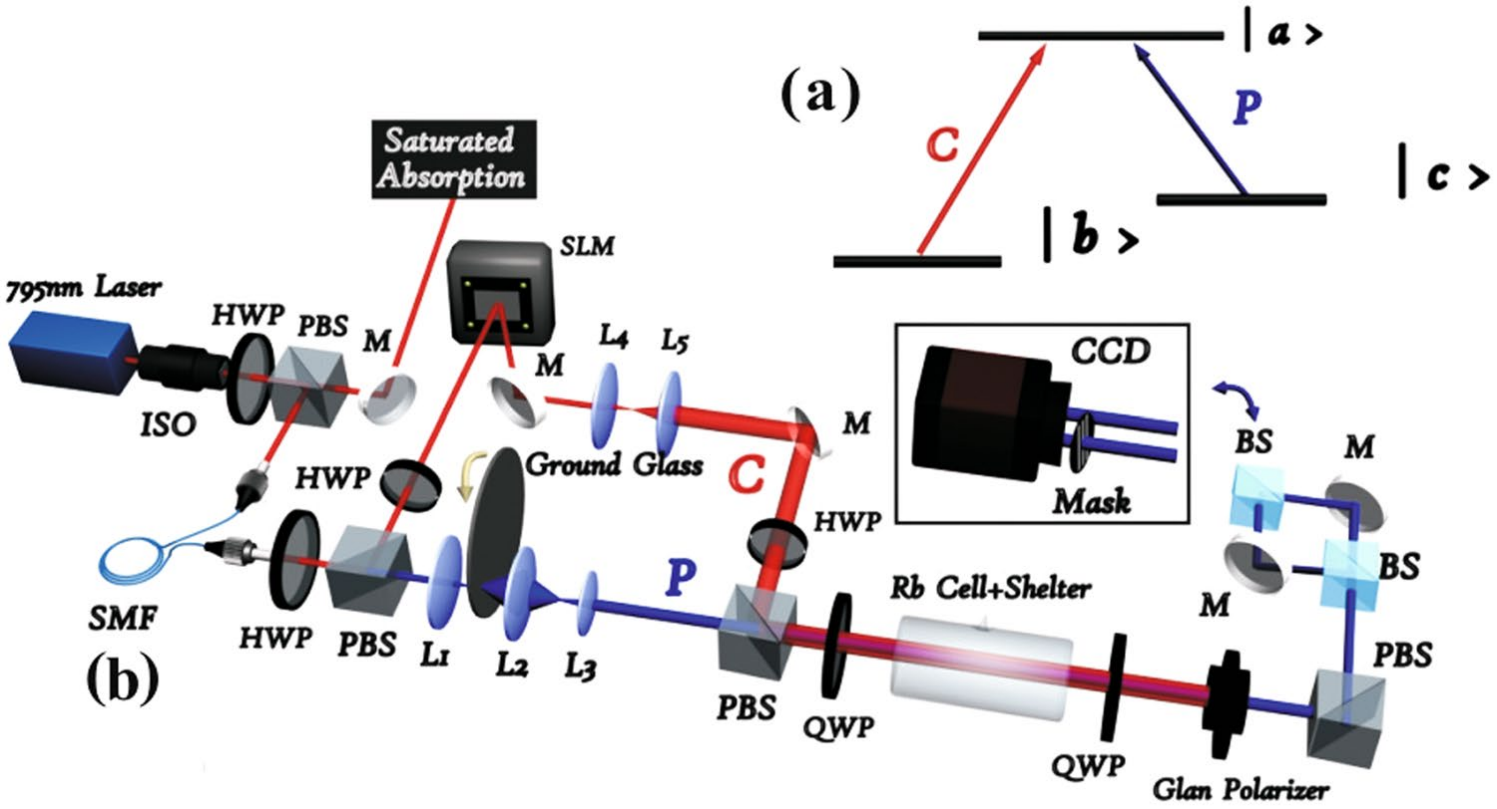
(**a**) Energy-level diagram of the CPT configuration. (**b**) Experimental setup. ISO: optical isolator; SMF: single mode fibre; SLM: spatial light modulator; L: lens; M: mirror; HWP: half-wave plate; QWP: quarter-wave plate; PBS: polarization beam splitter; BS: beam splitter; CCD: charge coupled device camera.

A typical CPT process in atomic medium is schematically shown in Fig. 1(a). The control (C) and probe (P) beams have orthogonal circular polarization and co-propagate with each other. In the experiment, we choose D1 transition of ^87^ *Rb* 5*S*_1/2_, *F* = 2 → 5*P*_1/2_, *F*′ = 1 to make a Λ type EIT scheme. As is shown in Fig. 1(a), |*a*〉, |*b*〉, |*c*〉 correspond to different Zeeman levels $|F^{\prime} =1,{M}_{F}=1\rangle$, $|F=2,{M}_{F}=2\rangle$ and $|F=2,{M}_{F}=0\rangle$, respectively. In the experiment, the control field was always much stronger than the probe field (${{\rm{\Omega }}}_{c}\gg {{\rm{\Omega }}}_{p}$), pushing most of the relevant atoms into the 5*S*_1/2_, *F* = 2, *M*_*F*_ = 0 Zeeman sub-level.

The schematic of the experimental setup is shown in Fig. 1(b). The laser beam was divided into two parts by a polarization beam splitter (PBS). One part was employed for frequency locking and was locked to the 5*S*_1/2_, *F* = 2 → 5*P*_1/2_, *F*′ = 1 transition. The other part was collected by a single mode fibre (SMF). In the same way, the beam from SMF with the waist width of approximately 3 mm was divided by a second PBS, where the horizontal polarization part was chosen as the probe field and the part with the vertical polarization served as the control field. For the control beam side, we used a computer-controlled liquid crystal spatial light modulator (Hamamatsu, x10468) (SLM), which relayed a holographic grating to generate an LG mode. Then, the generated LG beam was expanded to 18 mm by a telescope. For the probe beam, we selected the pseudo-thermal light as the probe; this was obtained by sending a Gaussian beam with horizontal polarization through a rotating ground glass, with the coherence time controlled by adjusting the velocity of the motor. In our experiment, the motor rotation speed is 2.5 r/min, corresponding to the coherence time of 20 ms for the pseudo-thermal light.

The probe and control beams combined on the PBS before the cell, which was arranged such that the expanded control beam and the pseudo-thermal light probe beam were overlapped in the Rb cell. The Rb cell length was 50 mm and was filled with enriched ^87^ *Rb* gas. A three-layer *μ*-metal magnetic shield was used to isolate the cell from ambient magnetic fields, while a solenoid inside the inner layer of the magnetic shield supplied an adjustable and longitudinal magnetic field. The temperature of the cell was set to 70 °C with a temperature controller. After passing through the cell, the probe and control beams were converted back to two orthogonal linear polarizations by two quarter-wave plates and were then separated by a PBS. Thus, we could record the spatial intensity distribution of the pseudo-thermal light by a CCD camera, as shown in the black box of Fig. 1(b). The pseudo-thermal light power was approximately 50 *μW* and that of the control beam was approximately 1.0 mW.

## Results and Discussion

**Figure 2:**
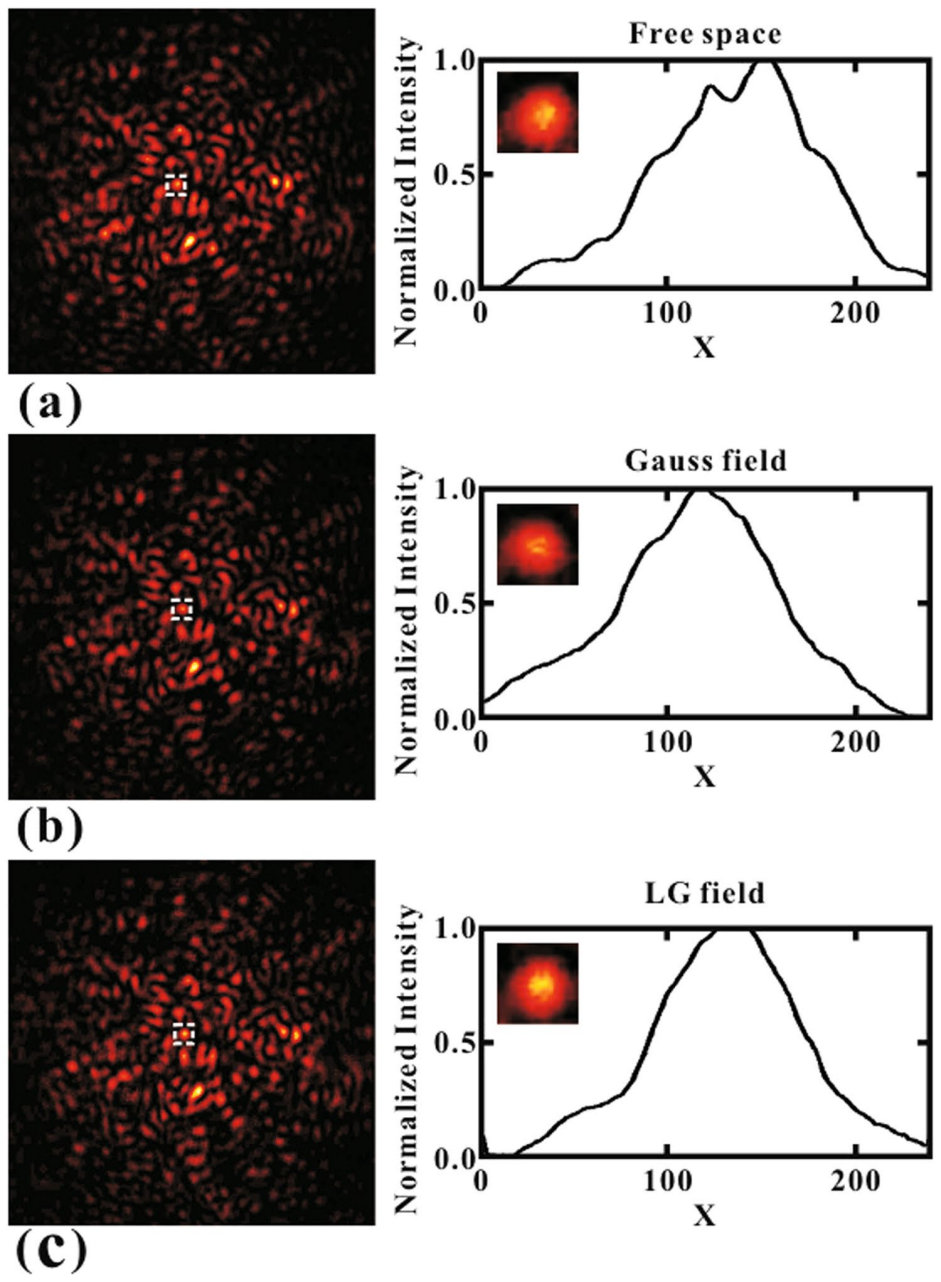
Experimental results of speckle compression for different cases. (Structured speckle less than average *l*_*c*_ is chosen). (**a**) Speckle pattern in free space, (**b**) Speckle pattern after the cell with Gaussian control beam, (**c**) Speckle pattern after the cell with LG control beam. Right column is the normalized transverse intensity distribution of one speckle, selected from the original and the modulated speckle patterns, respectively. The corresponding speckles FWHM are 0.176 mm in free space, 0.167 mm with Gauss field and 0.152 mm with LG field, respectively.

In CPT conditions, the speckle pattern that arrived through the atom cell was recorded by the CCD camera, and is shown in Fig. 2. In free space, the recorded pattern is shown in Fig. 2(a), while in the atomic case, the patterns are shown in Fig. 2(b,c). Based on the examination of Fig. 2(b), we found that upon the application of the Gaussian control beam, the speckle was compressed due to the atomic modulation effect, as discussed in ref. 15. We also replaced the Gaussian control beam to the LG beam with the quantum number *l* of 1. It is clear that the pattern from the atom cell obtained a much better image profile in this case. For a better comparison, we selected only one speckle from the original pattern, as shown in the right column of Fig. 2, and then we compared it with the corresponding speckle. We note that the right column profiles are the normalized transverse intensity distribution of the three speckles, which were generated by summing 20 pixel data at the central line of each speckle, and then calculating the average value. We could see that the line width becomes sharper when going from Fig. 2(a–c).

**Figure 3:**
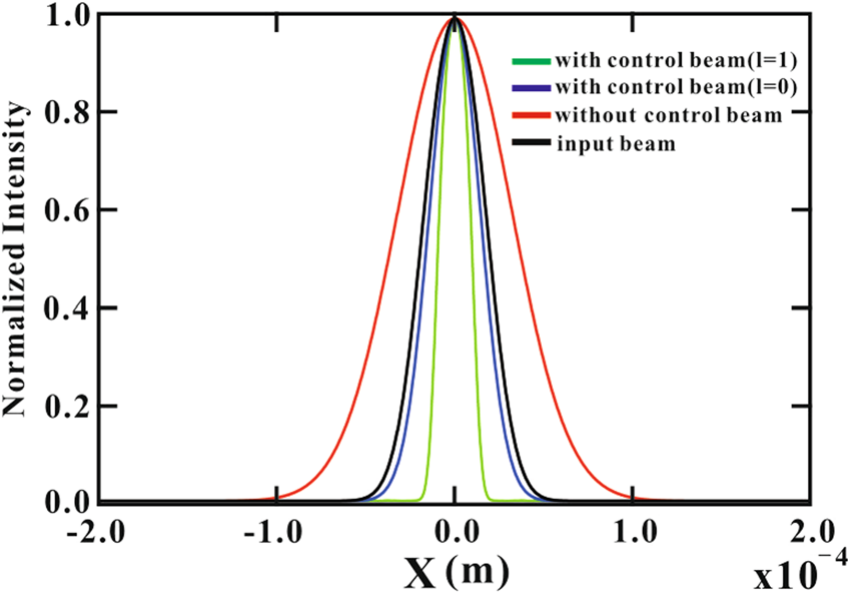
Width of the transmitted probe beam with normalized intensity. Colour profiles correspond to different control beams as shown in the figure.

Since the speckle was generated randomly after the ground glass, it was difficult to theoretically simulate the profile with different control fields. Therefore, we selected a Gaussian mode to serve as the probe $g={g}^{0}\,\exp \,[-{r}^{2}/2{\omega }_{0}^{2}]$, where $r=\sqrt{{x}^{2}+{y}^{2}}$ is the transverse spatial coordinate and *g* ^0^ is the input amplitude of the Gaussian beam. The wave equation of the probe beam propagating through the atomic medium in the CPT conditions can be expressed by Eq. (1).

1 $$\frac{\partial g}{\partial z}=\frac{ic}{2\omega }\,(\frac{{\partial }^{2}}{\partial {x}^{2}}+\frac{{\partial }^{2}}{\partial {y}^{2}})\,g+2i\pi k\chi g,$$

where *χ* is the susceptibility of the medium that depends on the transverse coordinates. In the simulation, we could solve the probe wave propagation equation numerically by using a higher-order split-step operator method. Meanwhile, we consider two spatial profiles for the strong control beam, of which the first one is a Gaussian beam with the spatial profile $G={G}^{0}\,\exp \,[-{r}^{2}/2{\omega }_{1}^{2}]$. The second one is the LG beam defined as $G={G}^{0}\sqrt{{r}^{2}/{\omega }_{1}^{2}}\,\exp \,[-{r}^{2}/2{\omega }_{1}^{2}]$. Here *G* ^0^ is the peak amplitude, and *ω*_1_ is the spatial width of the control field. The results obtained using Eq. (1) are shown in Fig. 3. We can compare the widths of the probe at the output for different control beams. Clearly, the control beam with the LG mode gives a much narrower beam width of the probe.

The absorption and diffraction of the probe beam are very sensitive to the intensity of the control beam. Classically, if the laser frequency was tuned to a value far away from the atomic transition, the speckle pattern would be significantly distorted after propagating through a vapor cell, because this distortion mostly arises from the atomic diffusion in propagation. However, in the CPT conditions, the spatial profile of the strong control beam makes the medium inhomogeneous along the transverse direction, leading to absorption and dispersion of the medium being crucially dependent on the intensity and shape of the strong control beam. In our experiment, we observed that the speckled pattern was compressed effectively in the presence of the Gaussian beam control and LG beam control spatial profiles. Physically, the diffraction elimination effect can also be thought of as a kind of deflection effect, as has been observed by Firstenberg *et al*.^5, 6^. The speckle beam “finds” the control beam and refracts in its direction, regardless of the original direction of the speckle. Thus, all transverse momentum components of an image refract into the same direction and traverse the cell together, maintaining their phase relation. On exiting the cell, each component returns to its original direction. Compared to the Gaussian control beam, LG beam produced a relatively higher energy in a given transverse area and showed uniform intensity at the top. This leads to higher transmission for the probe beam because the absorption and diffraction of the probe beam are very sensitive to the intensity.

To obtain a better theoretical understanding for resolution enhancement of GI in our experiment, we define the second-order GI, as depicted in ref. 23,

2 $${G}^{\mathrm{(2)}}({r}_{R})={T}^{2}({r}_{R})\otimes som{b}^{2}\,(\frac{\pi D}{\lambda d}|{r}_{T}-{r}_{R}|),$$

where *r*_*O*_(*O* = *T*, *R*) are the centre coordinates on the detection plane, and *T* ^2^(*r*_*R*_) is the object function. We can see that two terms are present as shown in Eq. (2). For the first term, we can consider the object function *T* ^2^(*r*_*R*_) as a probe. After the integral of the whole object plane, we obtain the convolution in spatial dimension as the bucket value. For a speckle, its full width at half maximum (FWHM) is $2\sqrt{2ln2}{\sigma }_{g}\propto \tfrac{\lambda d}{D}$, *σ*_*g*_ is the average grain size of the ground glass, which is several tens *μ*m in our experiment. Therefore, it is clear that the resolution is mainly determined by the FWHM of the speckle.

**Figure 4:**
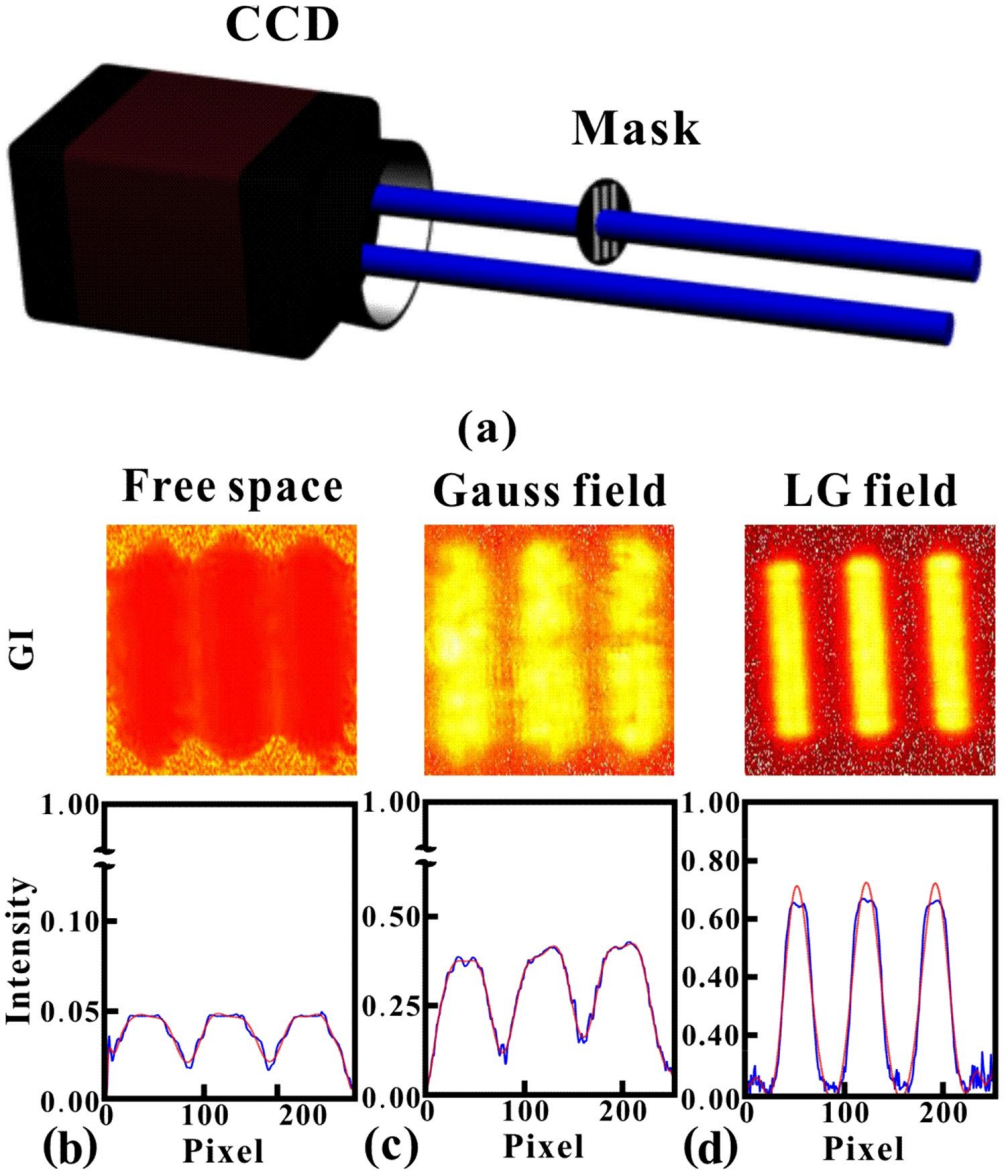
The GI experiment results under three cases: free space, Gaussian control beam and LG control beam. (**a**) Detection setup for GI: the object (three slits mask) is placed on one beam, which is recorded by one half of the CCD camera, the other reference beam is recorded by the second half. The GI results and the corresponding image line shape are present from (**b**–**d**). In three different cases, the FWHM of the image line shape is 0.59 mm, 0.41 mm and 0.21 mm respectively. And the corresponding image contrast is 0.48, 0.65 and 0.97.

Using the obtained speckles, we performed the corresponding GI experiments. A three-slits mask was placed on one arm of the speckle beam, another arm was used as the reference beam, and both of these are recorded by the CCD camera shown in Fig. 4(a). The results are presented in Fig. 4(b–d), showing the reconstructed images of the mask and intensity distributions of the experiment results. Figure 4(b) shows the GI results of the case in free space. Obviously, it is very difficult to distinguish the three slits. However, when applying the Gaussian control beam that passes through the atom vapor, the three slits can be very clearly distinguished, as shown in Fig. 4(c); this means that we obtained a much better resolution of GI in atom vapor. Moreover, the GI resolution became even better with the LG control beam, as presented in Fig. 4(d).

Actually, such good resolution is quite reasonable. Normally, for a speckle, the transverse coherent length (*l*_*c*_) is determined by both the grain size of the ground glass, the glass rotation speed and the original coherent length of the laser source. However, we found it is also possible to compress the speckle with atomic medium to obtain smaller *l*_*c*_ in the experiment. As is shown in ref. 15, the GI resolution strongly relies on *l*_*c*_, speckles with smaller *l*_*c*_ are expected to have better resolution. In the experiment, the mask is a transparent three slits with full width of 1.8 mm, and the width of each single slit is 0.3 mm. By contrast, average *l*_*c*_ of the pseudo-thermal source is measured about 0.35 mm in our experiment, that is why giving rise to the non-distinguishable GI shown in Fig. 4(b). However, after applying the control beam in the atomic cell, the spatial modulated medium susceptibility made *l*_*c*_ smaller than that in free space. In the experiment, the more the speckle was compressed, the smaller FWHM of speckle would be. According to the theoretical analysis in Eq. 2, smaller FWHM will make the image resolution better. Apparently, the speckle was compressed most effectively, when the control beam was changed to the LG mode, as shown in Fig. 2(c). For the LG mode, the “donuts” shape transverse intensity made the atom medium more inhomogeneous along the transverse direction, leading to absorption and dispersion of the speckle pattern being crucially dependent on propagation. From the calculation results presented in Fig. 3, it is obvious that width of the probe at the output was compressed most effectively in the present of the LG control beam, and that is why we can observe the best resolution GI with the LG control beam. To have a better comparison of the experimental results obtained for different cases, we defined the image contrast ratio ${\mathcal R}$ for image evaluation,

3 $$ {\mathcal R} =\frac{{I}_{max}-{I}_{min}}{{I}_{max}+{I}_{min}}.$$

Since the image in free space is almost undistinguishable, there was no reason that the image contrast was only approximately 0.48. Then, the value was improved to 0.65 after the application of the Gauss field control beam, and it is much easier to see the image of the three slits. Finally, the image contrast reached 0.97 with LG control beam, and the image was improved to nearly the same level as the initial slit. The current image contrast is nearly 1.5 times better than that of our previous work ref. 15. Moreover, from the image profile shown in Fig. 4(b–d), we can find that the FWHM of the image profile was also greatly compressed with the transverse control beam, substantially improving the image resolution.

**Figure 5:**
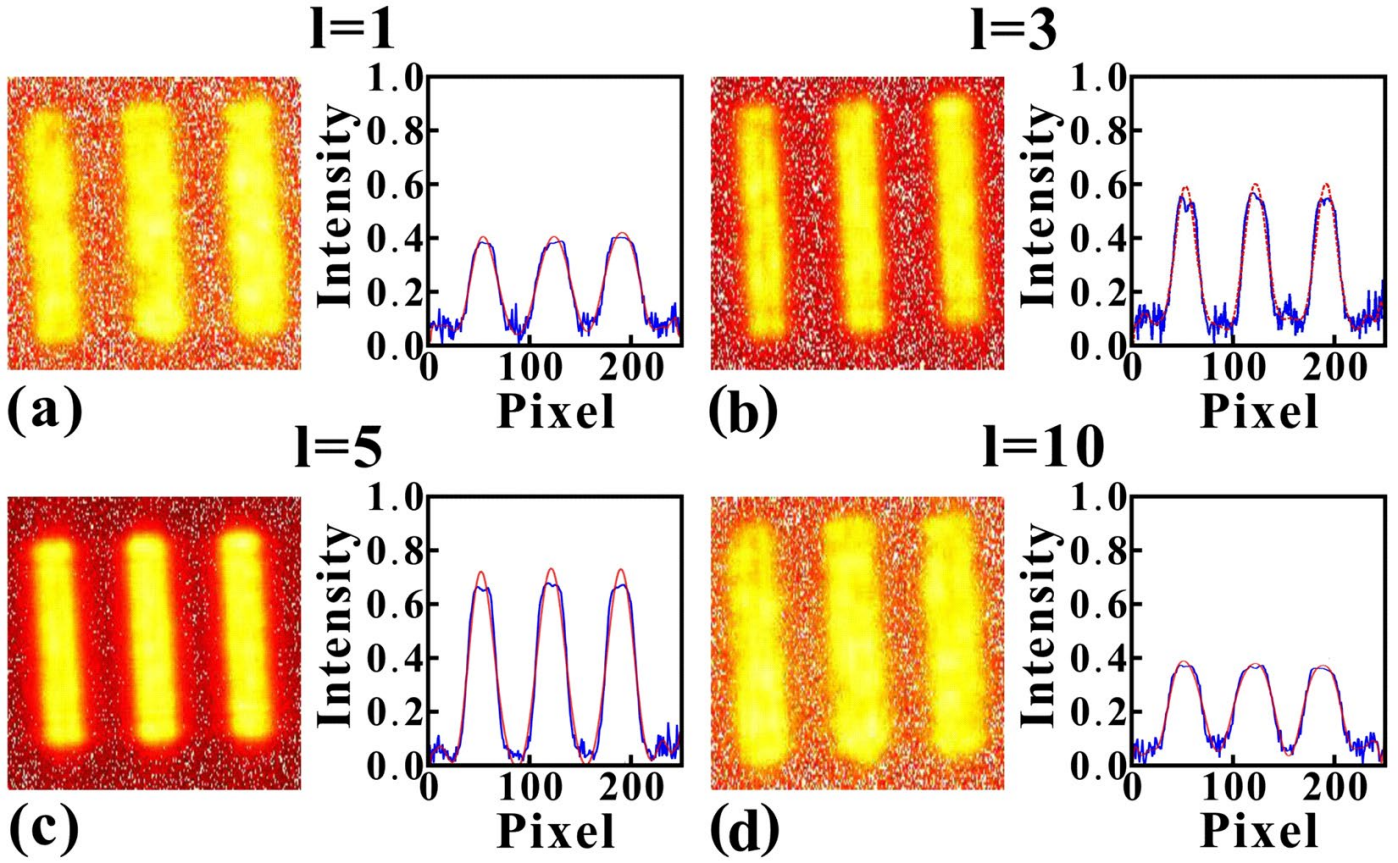
GI results with different charges of LG control beam. (**a**–**d**) are the results for control LG mode with l = 1, 3, 5 and 10, respectively. From (**a**–**d**), the FWHM of the image line shape is 0.27 mm, 0.20 mm, 0.21 mm and 0.28 mm respectively, and the corresponding image contrast is 0.88, 0.94, 0.97 and 0.74.

Inspection of Fig. 4 shows that the GI resolution greatly relies on the spatial profile of the control beam, because the FWHM of the speckle was crucially dependent on the absorption and dispersion of the inhomogeneous medium along the transverse direction, which can be manipulated by the spatial profile of the strong control beam. To obtain a clear understanding of the relationship between the LG control beam and the GI resolution, we generated four different LG beams to serve as the control beam, with values of *l* = 1, 3, 5 and 10, respectively. Actually, the FWHM of the speckle could be manipulated by changing different experimental parameters, such as control beam power, frequency detuning, and atomic density as has been discussed in ref. 15. Here, we are mainly concerned with the GI stability against LG charge. In this experiment, the probe beam power was always 40 *μ*W, and the power values of the control beams for different LG modes were approximately 1 mW. The detuning for the control and probe beam was also maintained during the experiment. The results are displayed in Fig. 5, where (a–d) show the cases for the LG mode with *l* = 1, 3, 5 and 10, respectively. Examination of Fig. 5(a–c) shows that the resolution of GI was improved with the increase in the number *l*, that is because the control beam intensity profile was getting sharper with lager *l* number, which made the speckles compressed more. Comparing with the FWHM at *l* = 3 and in free space, we found that the ghost image resolution can be increased by factor 3, and the image contrast reached 0.94. However, when the *l* reached as high as 10, the diameter of the “donuts” shape was approximately 4 mm, which already exceeded the probe beam size. This condition gave rise to a nearly complete absence of overlap between the control beam and probe beam. In this case, there was not enough control power to ensure the manipulation in the medium along the transverse, which may result in a worse resolution when calculating the second-order correlation imaging. If the size of the probe beam is increased further, the LG number *l* for the best resolution condition will certainly increase. It is important to mention that our experimental setup was limited by the fixed speckle size, and it was very difficult to tune the speckle pattern size continuously. Further extension of such a compression experiment would be more applicable if the speckle size can be set to arbitrary value by using lens pairs and some other methods.

## Conclusion

In conclusion, we reported on an experimental realization of GI resolution enhancement through the CPT effect. The results clearly show that the GI resolution is related to the *l*_*c*_ of the speckle pattern, which could be modulated by different transverse control beam profiles in atomic vapor. Together with the recent experimental results on pseudo-thermal light speckle modulation in atomic medium, our result of preservation of the spatial multimode correlation in the EIT medium strongly implies the possibility of realization of multimode modulation in nonlinear processes. We therefore believe that the GI in atom vapor demonstrated in this work will open up important new applications of quantum and classical correlation imaging, image processing, biomedical imaging, and other imaging techniques.
